# Phyto-synthesized facile Pd/NiOPdO ternary nanocomposite for electrochemical supercapacitor applications†

**DOI:** 10.1039/d2ra07292k

**Published:** 2022-12-12

**Authors:** Irum Shaheen, Khuram Shahzad Ahmad, Camila Zequine, Ram K. Gupta, Andrew G. Thomas, Anjum Qureshi, Mohammad Azad Malik, Javed H. Niazi

**Affiliations:** Department of Environmental Sciences, Fatima Jinnah Women University Rawalpindi Pakistan chemist.phd33@yahoo.com dr.k.s.ahmad@fjwu.edu.pk; Department of Chemistry, Pittsburg State University 1701 South Broadway Street Pittsburg KS 66762 USA; Department of Materials, Photon Science Institute, Sir Henry Royce Institute, University of Manchester Alan Turing Building, Oxford Road Manchester M13 9PL UK; SUNUM Nanotechnology Research, and Application Center, Sabanci University Orta Mah., Tuzla 34956 Istanbul Turkey

## Abstract

Sustainable and effective electrochemical materials for supercapacitors are greatly needed for solving the global problems of energy storage. In this regard, a facile nanocomposite of Pd/NiOPdO was synthesized using foliar phyto eco-friendly agents and examined as an electrochemical electrode active material for supercapacitor application. The nanocomposite showed a mixed phase of a ternary nano metal oxide phase of rhombohedral NiO and tetragonal PdO confirmed by X-ray diffraction (XRD), scanning electron microscopy (SEM) and XPS (X-rays photoelectron spectroscopy). The optical (direct) energy value of the synthesized nanocomposite was 3.14 eV. The phyto-functionalized nanocomposite was studied for electrochemical supercapacitor properties and revealed a specific capacitance of 88 F g^−1^ and low internal resistance of 0.8 Ω. The nanoscale and phyto organic species functionalized nanocomposite exhibited enhanced electrochemical properties for supercapacitor application.

## Abstract 2

The natural phyto bio-factories were successfully utilized for the cost-effective synthesis of facile Pd/NiOPdO ternary nanocomposite for energy storage application with enhanced electro-active site.

## Graphical abstract

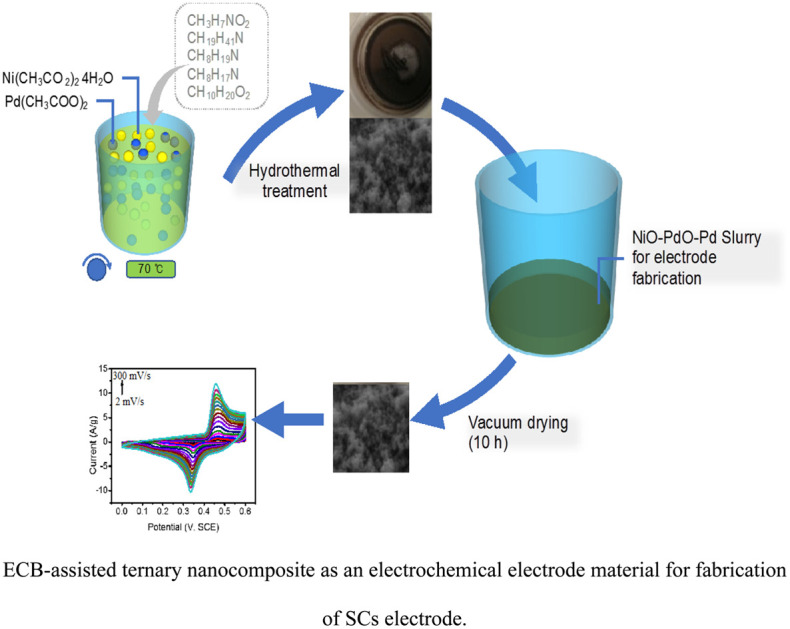

## 1. Introduction

In the era of industrial advancement and technology, the global world is facing two major environmental crises of depleting fossil fuels and increasing environmental pollution.^1,2^ The concern is not limited to fossil fuel-based non-renewable energy resources but also their undesirable environmental effects.^3,4^ The depletion of fossil fuels and their environmental effects have increased the dependency of human society on clean renewable energies such as solar energy. Nevertheless, renewable energies demand sustainable and efficient energy storage systems to ensure efficient, uninterrupted, affordable, and reliable energy supplies^5,6^ Consequently, there is a serious need for sustainable and efficient systems for energy storage. Among various energy storage systems, supercapacitors (SCs) are considered as the most promising candidates having noteworthy properties such as lower maintenance cost, safer operation, and excellent response.^7,8^ It is also well known that the performance of the SCs is critically dependent on their electrode active materials. The overall cost of SCs mainly depends on their electrode material. To develop economic SCs for energy storage, there is a need to reduce the cost of electrode materials.^9^ Therefore, the present study is an attempt to develop lower-cost and sustainable electrode materials for SCs.

Metal oxides are highly studied and adopted materials for SCs because of their environmentally friendly nature, lower cost, and being earth-abundant.^10,11^ metal oxides are the preferred electrode material for SCs, as these possess high energy density than carbon materials and higher electro-chemical stability than conducting polymers (CPs). Moreover, metal oxide electrodes have characteristic features of both carbon material and faradaic materials. The metal oxides can store energy electrostatically like carbon materials and exhibit faradaic redox reactions as those of CPs. Metal oxides reveal faster redox reactions between the active material and the electrolyte because of their variable oxidation states.^12,13^ Therefore, it is necessary to synthesize metal oxide nanomaterials using a cost-effective method, which could be used as efficient redox materials to store energy. One such synthesis route is the biogenic synthesis route. Biogenic synthesis is a cost-effective and efficient method for the synthesis of nanomaterials which has reduced the overall cost of the energy material as well as a green approach in comparison to other physicochemical synthetic routes.^14,15^

In the present study, a novel Pd/NiOPdO ternary nanocomposite was synthesized by the sustainable phyto-synthetic route without using any additional chemicals and reagents. As an alternative to chemical conventional synthesis methods, plant-mediated synthesis is considered safe, cost-effective, and environmentally sound for the synthesis of a wide range of nanomaterials. PdO and NiO are among the most competent metal oxides for electrochemical energy storage applications. However, the higher cost of these faradaic materials and the cost associated with their synthesis limit their application in sustainable lower-cost energy storage materials. In the current work, PdO and NiO were synthesized by the lower-cost synthesis route without using any cost reagent or chemical. The only used chemicals here are precursor salts of palladium acetate [Pd(CH_3_COO)_2_] and nickel acetate tetrahydrate [Ni(CH_3_CO_2_)_2_·4H_2_O] for the synthesis of PdO and NiO using *Euphorbia cognata Boiss* phyto extracted reagents. The cost of Pd/PdO/NiO electrode material was controlled by adopting lower-cost sustainable synthesis routes. The plant-mediated biogenic synthesis of stable metal oxides nanomaterials is reported and here phytochemicals are not only used as reducing agents but also used as stabilizing agents of the metal oxide nanocomposite.^16^ In the present phyto-synthesis, the chemical compounds of *Euphorbia cognata Boiss* (ECB) leaves were extracted and used as fuel in the synthesis of Pd/NiOPdO nanomaterial. The ECB leaves are reported to be medicinally significant leaves and were specifically selected due to the fact that this plant is reported to have different phenolics and flavonoid compounds that are responsible for their medicinal and antioxidant activities. The antioxidant effects of phenolic compounds are due to their redox properties and are attributed to various mechanisms such as free radical scavenging, transition-metal chelating, and oxygen-quenching capacity.^17,18^ Thus, medicinal plant leaves of ECB were selected plant in order to ensure that ECB leaves have significant bio-molecules for the synthesis of nanomaterials.

Keeping in view the above-mentioned significance of ECB, herein, we have reported the synthesis of ternary nanocomposite Pd/NiOPdO for the first time in the current study which was then used in the fabrication of a supercapacitor electrode. The electrochemical properties of newly synthesized material were studied at a range of scanning rates by cyclic voltammetry (CV), and at verifying current densities by galvanostatic charge–discharge (GCD). A moderate specific capacitance of 88 F g^−1^ was observed for Pd/NiOPdO nanocomposite while lower internal resistance of 0.8 Ω was determined by electrochemical impedance spectroscopy (EIS). Consequently, the current work has demonstrated that natural bio-factories of ECB were successfully utilized for the cost-effective synthesis of sustainable novel ternary nanocomposite for electrochemical energy storage applications.

## 2. Experimental details

### 2.1 Material and methods

Palladium acetate [Pd(CH_3_COO)_2_], nickel acetate tetrahydrate [Ni(CH_3_CO_2_)_2_·4H_2_O], ethanol (C_2_H_5_OH), methanol (CH_3_OH), acetylene black, polyvinylidene fluoride (PVDF) and *N*-methyl pyrrolidinone (NMP). All chemicals were provided by Sigma Aldrich, Germany.

### 2.2 Synthesis of metal oxide nanocomposite

**Scheme 1:**
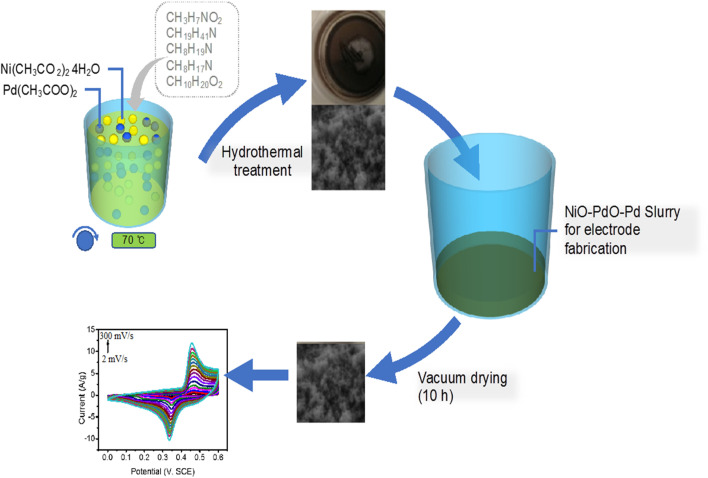
The schematic illustration of ECB-assisted ternary nanocomposite as an electrochemical electrode material for fabrication of SCs electrode.

The *Euphorbia cognata Boiss* foliar extract (ECFE) was prepared by modified reported methodology^19,20^ which is illustrated in Scheme 1. Briefly, 10 mL of ECFE was mixed with Pd(CH_3_COO)_2_, (20 mM) under continuous stirring for 10–15 min and 10 mL of ECFE was mixed with 20 mM of Ni(CH_3_CO_2_)_2_ aqueous solution on same stirring conditions. After 15 minutes both solutions were mixed and stirred for 2 h at 70 °C. Then, the solution was evaporated at 95 °C followed by annealing inside an air furnace at 450 °C for 4 h to obtain phyto-functionalized Pd/NiOPdO nanocomposite.

### 2.3 Characterization

Pd/NiOPdO nanocomposite was analyzed by UV-Vis spectrophotometer (1602 – Biomedical Services Spain (UV-Vis)), XRD (XRD5 PANalytical X'Pert Pro, in the School of Materials, University of Manchester (UoM)) and XPS (Kratos-Axis Ultra spectrometer XPS). The organic functional groups/bonds in the synthesized materials were confirmed *via* FT-IR spectroscopy (FTIR, 8400, Shimadzu, Japan) and gas chromatography-mass spectrometer (GCMS QP5050). The surface morphology was studied by SEM (Quanta 250-FEG, Thermo Fisher Scientific USA, having EDX Bruker, USA in UoM, UK).

### 2.4 Electrochemical characterization

The phyto-synthesized nanocomposites based SCs electrode material was prepared using a slurry that was comprised of Pd/NiOPdO nanocomposite (4 mg), acetylene (carbon) black (1 mg) and PVDF (1 mg) in *N*-methyl pyrrolidinone solvent. The electrochemical active metal oxide electrode material deposited on Ni foam was used as substrate and followed by thermal treatment. The selection of Ni foam was made based on the fact the Ni foam substrate is relatively inactive in the electrochemical experimentations by showing CV curves of the negligible integrated area and peak current density. Ni foam has no contribution to the capacitance of the electrode.^5,6,19,20^ The mass of active material on the electrode was 4 mg which was carefully measured after and before the preparation pf electrode by analytical laboratory weighing balance. The electrochemical investigation of as-synthesized nanocomposite electrodes was carried out by cyclic voltammetry (CV), galvanostatic charge–discharge (GCD), and electrochemical impedance spectroscopy (EIS). CV, GCD, and EIS were studied in a three-electrode workstation using KOH (3 M) as an electrolyte. The CV was performed at scan rate ranging from 2 to 300 mV s^−1^ and GCD was carried out at different current densities from 0.5 to 30 A g^−1^ while EIS analysis was studied at 50 mHz to 10 kHz frequency range.

## 3. Results and discussion

### 3.1 Structural and optical properties of Pd/NiOPdO nanocomposite

Phytogenic Pd/NiOPdO nanocomposite was synthesized using foliar ECB extract.

**Fig. 1:**
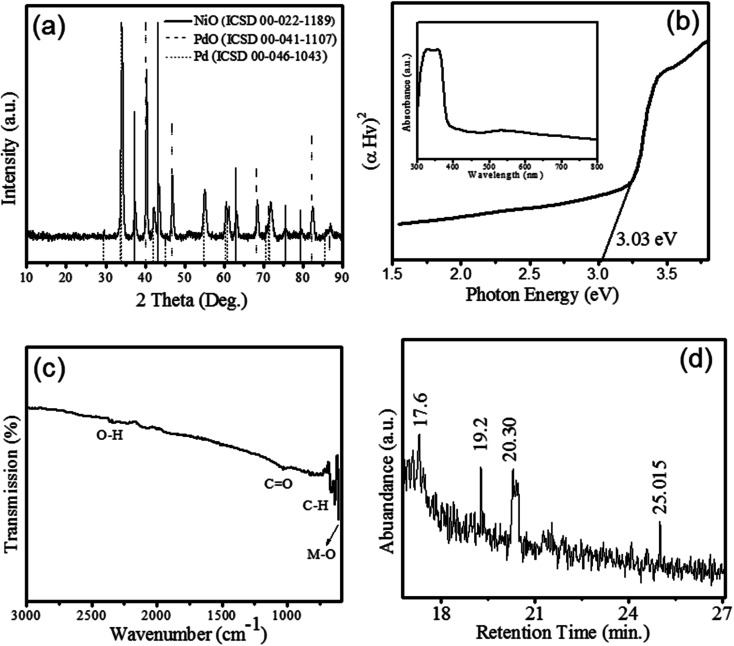
(a) The X-rays diffraction patterns of ECFE-assisted Pd/NiOPdO nanocomposite. (b) The Tauc’s plot with the optical energy value of Pd/NiOPdO (c) FTIR spectra of Pd/NiOPdO and (d) GC-MS spectra of the phyto-stabilizing Pd/NiOPdO nanocomposite.

The XRD patterns of NiO (ICSD 00-022-1189), Pd (ICSD 00-046-1043) and PdO (ICSD 00-041-1107) is shown in figure. The XRD patterns revealed the growth of rhombohedral-shaped NiO with space group of *P*6_3_*mc* and produce prominent peaks depicted by (*) at 2theta (*θ*) = 37.42°, 43.47°, 63.04°, 75.6°, and 79.54° corresponding to (003), (012), (110), (021), and (006) *hkl* planes respectively. Fig. 1a revealed the XRD pattern of PdO indicated (♦) at 29.55°, 34.11°, 42.2°, 55.06°, 60.53°, 61.11°, and 71.76° corresponding to (100), (101), (110), (112), (103), (200), and (211) lattice reflection planes respectively. The identified space group of PdO is *P*4_2_/*mc* with a tetragonal shape. Moreover, the pattern peaks of Pd (●) were observed at 2theta (*θ*) = 40.3, 46.86, 68.38, 82.38, and 86.88 corresponding (111), (200), (220), (311), and (222) *hkl* planes, respectively. Therefore, XRD pattern clearly showed the formation of nanocomposite with a mixed phase of electrochemically active oxides of NiO and PdO as Pd/NiOPdO. The size of Pd/NiOPdO crystallite was determined by Debye Scherrer's equation^21^ and calculated as 21 nm ± 3. The UV-Vis spectrum of Pd/NiOPdO nanocomposite is shown in Fig. 1b and the inset shows the two absorption bands corresponding to organics and oxides respectively. Fig. 1b is a plot of the optical-direct energy of Pd/NiOPdO (Tauc plot) and an optical band of nanocomposite found to be 3.14 eV.

The presence of functional groups of *Euphorbia cognata Boiss* foliar on Pd/NiOPdO was confirmed by FTIR and GCMS and shown in Fig. 1c and d. The presence of prominent vibrational peaks at 2337 cm^−1^, 1026 cm^−1^, 1200–1700 cm^−1^, and 810 cm^−1^ correspond to O–H, amide region, C=O and C–H stretching vibrations, respectively. The presence of metal and carbon/oxygen in the form of M–C or M–O (M = Ni/Pd) was observed between 600 and 400 cm^−1^. The functional groups of foliar extract were observed at 659.69 cm^−1^, 638.9 cm^−1^ and 610.55 cm^−1^ (Fig. 1c) which corresponds to aromatics and amine groups in the synthesized nanocomposite.^22,23^ The GC-MS spectra in Fig. 1d is showing peaks at different retention times (min) of 17.6, 19.2, 20.30, and 25.01. These peaks in the GC chromatogram showed the presence of octodrine (C_8_H_19_N), and cyclobutanol (C_4_H_8_O) species on Pd/NiOPdO nanocomposite **(**according to the NIST library). Therefore, GCMS and FTIR results successfully confirmed the cyclobutanol and octodrine group presence in Pd/NiOPdO nanocomposite.

**Fig. 2:**
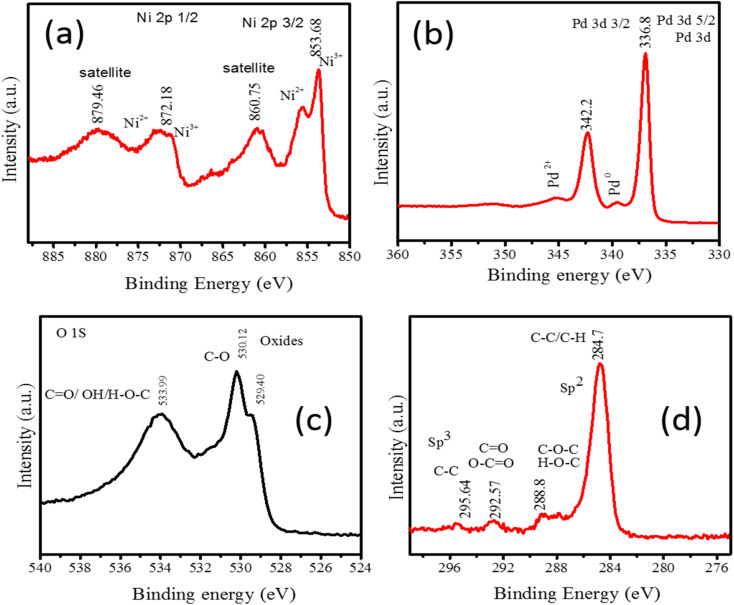
XPS core level spectrum of Pd/NiOPdO nanocomposite (a) Ni 2p, (b) Pd 3d, (c) O 1s, and (d) C 1s.

The XPS spectra of Pd/NiOPdO nanocomposite are shown in Fig. 2a–d. The peaks corresponding to Ni 2p_3/2_ (853.86 eV) and Ni 2p_1/2_ (872.17 eV) are observed in Fig. 2a. The regions at 860.75 eV and at 872.1 eV are the satellite regions having Ni^2+^/Ni^3^.^20^Fig. 2b is presenting the core spectrum of Pd 3d having Pd 3d_5/2_ and Pd 3d_3/2_. The O 1s region of Pd/NiO/PdO has been presented in Fig. 2c which shows the peaks fitting at binding energies of 529.40 eV, 530.12 eV, and 533.99 eV corresponding to the oxide. C=O and C–OH respectively.^19,20^ The C=O and C–OH species are due to phytochemicals of the ECFE with little contribution from adventitious hydrocarbons. The C 1s in Fig. 2d is presenting the peaks at 284.7 eV due to adventitious C–C and C–H. The peak at 288.8 eV is related to the C–OH and C=O of bioactive compounds in addition to adventitious hydrocarbon. However, the two regions above 290 eV at binding energies of 292.57 eV and 295.64 eV have originated from the carbon-containing species of plant extract. However, these two peaks at binding energies of 293.2 eV and 296.4 eV are also due to the spin–orbit split K 2p peaks, suggesting a small amount of contamination due to the organic molecules of the leaves.^19,20^ The C-derived peaks in Fig. 2d are consistent with the stabilizing molecules of the plant extract above 290 eV while the region below 290 eV has minor adventitious carbon contribution along with phytocompounds. The O 1s core is revealed in Fig. 2c and confirms the oxides and organic carbon presence of phyto extract.^19,20^ The organic groups were also evident with the appearance of C 1s core spectrum in Fig. 2d.^20–22^ The additional peak in O 1s spectra (at 533.99 eV) and in the C 1s spectra (above 290 eV) reveal the surface tailoring of Pd/NiOPdO due to the ECFE. Therefore, XPS results demonstrated ECFE functionalized and stabilized Pd/NiOPdO nanocomposite.

**Fig. 3:**
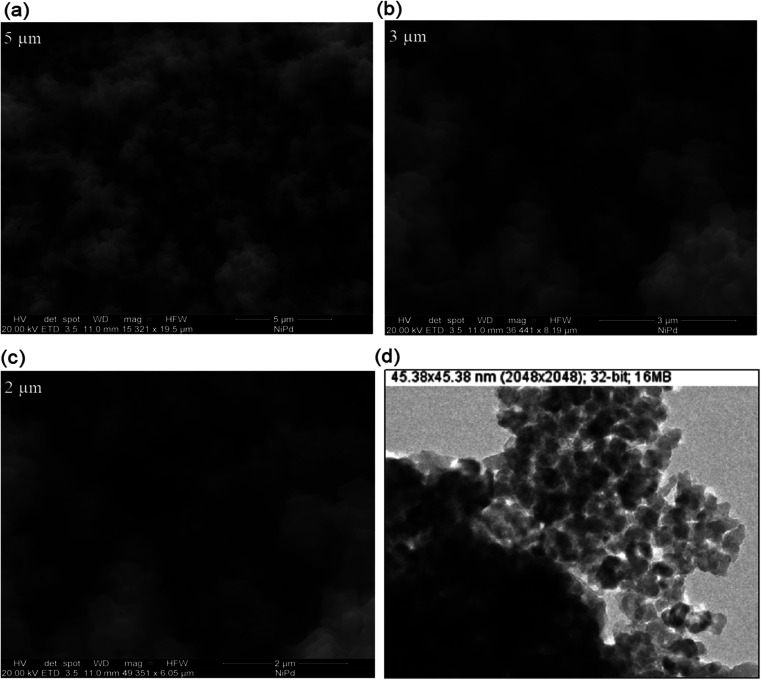
(a)–(c) The SEM images of showing the surface morphology of ECFE-assisted synthesized Pd/NiOPdO nanocomposite at different magnifications, and (d) TEM image of ECFE-assisted Pd/NiOPdO nanocomposite.

The SEM images of as-synthesized Pd/NiOPdO nanocomposite are presented in Fig. 3a–c. The SEM demonstrated the spherical-shaped particles at 5 μm, 3 μm, and 2 μm in Fig. 3a–c and at 153 21×, 364 41×, and 493 51× magnifications respectively. Due to the presence of mixed phases *i.e.*, Pd/PdO along with NiO, individual particle presences and particle distribution were not clearly shown by FESEM, therefore, TEM analysis was carried out as presented in Fig. 3d. In consistency with SEM morphological examination, the TEM image in Fig. 3d is illustrating spherical shapes of synthesized Pd/NiOPdO nanocomposite at ∼50 nm. Furthermore, the TEM image also shows voids or spaces among particles indicating less agglomeration among particles. The less agglomeration by TEM is suggesting the role of foliar organic agent as a reducing and stabilizing agent as described in published literature.^20–23^

**Fig. 4:**
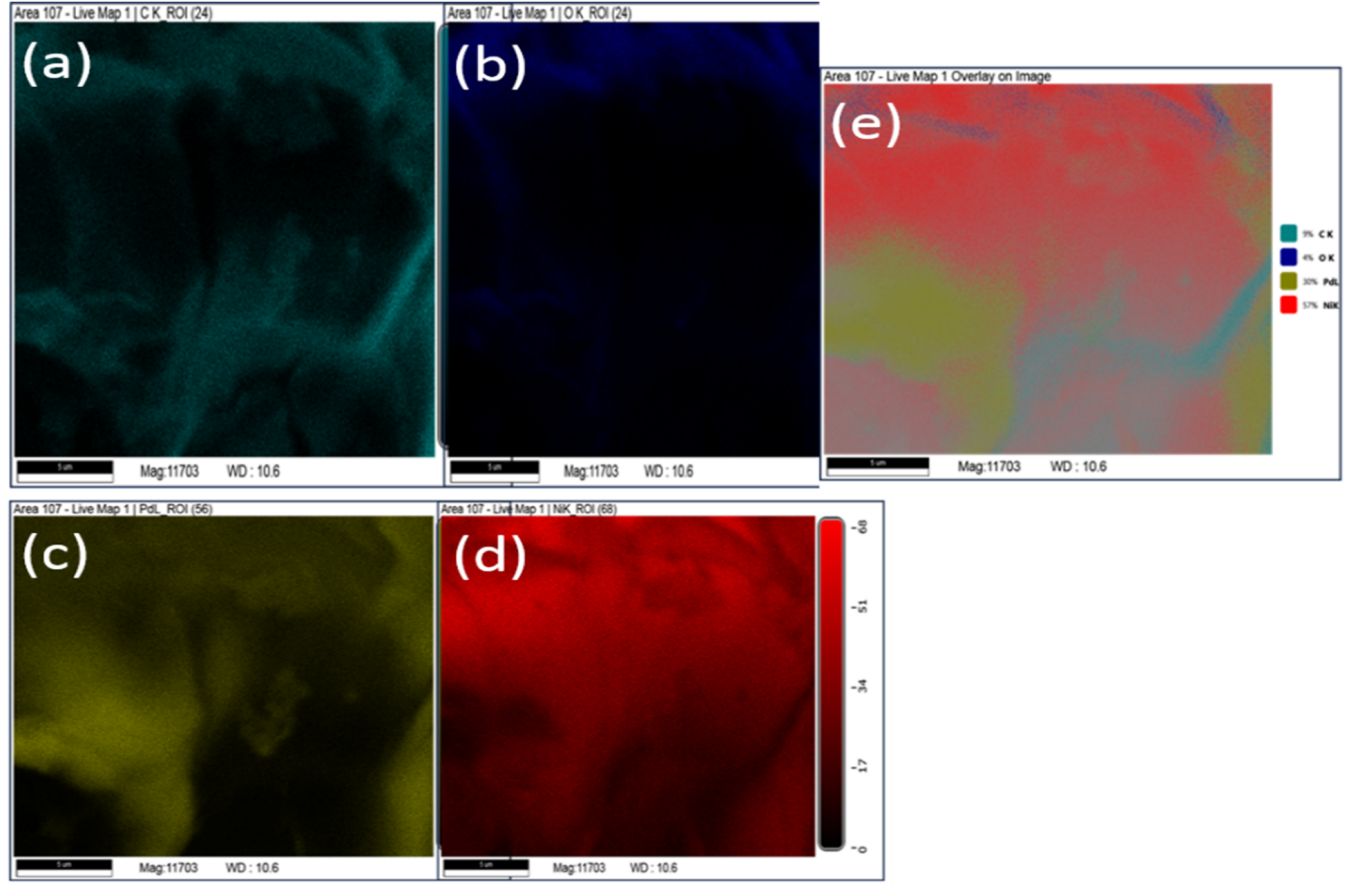
The EDX mapping of phyto synthesized Pd/NiOPdO nanocomposite shows elemental (a) carbon, (b) oxygen, (c) Pd, (d) Ni and (e) overlay EDX map.

Further, elemental analysis of Pd/NiOPdO nanocomposite is shown in Fig. 4a–e. The presence of carbon, oxygen, Pd, Ni clearly showed in the EDX map images (Fig. 4a–d) and overlay image (Fig. 4e) of Pd/NiOPdO nanocomposite.

### 3.2 Supercapacitor measurements

**Fig. 5:**
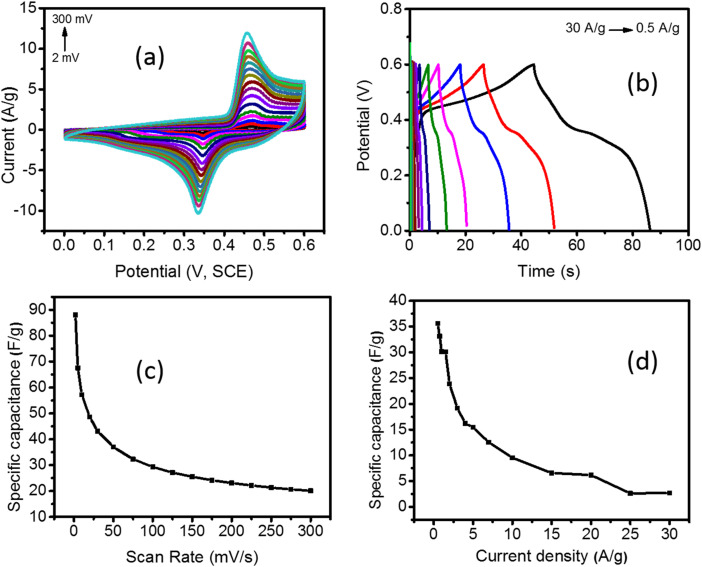
(a) The CV response of Pd/NiOPdO nanocomposite electrode at different scan rates, (b) GCD profile of Pd/NiOPdO nanocomposite electrode at different current densities, (c) the specific capacitance measured with Pd/NiOPdO SCs electrode at different scan rates and, (d) the specific capacitance measured with Pd/NiOPdO SCs electrode at current densities.

**Fig. 6:**
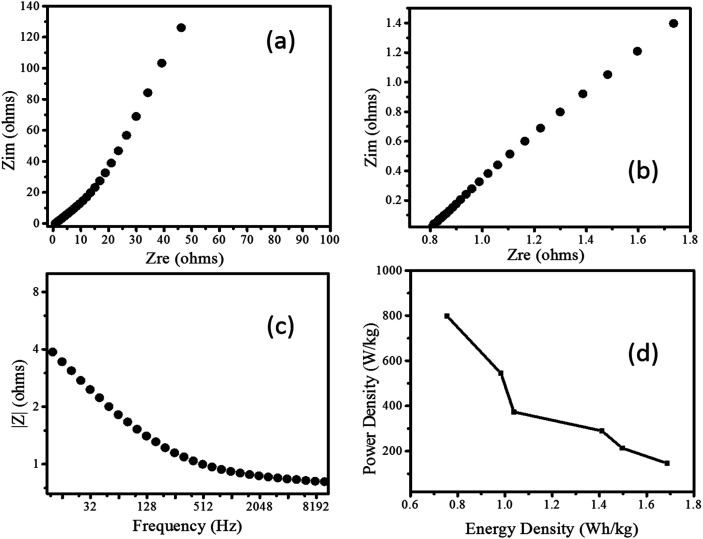
The Nyquist plot of Pd/NiOPdO nanocomposite showing imaginary impedance as a function of real impedance at (a) low frequency and (b) high frequency. (c) The magnitude of impedance as a function of applied AC frequency and, (d) Ragone plot of Pd/NiOPdO nanocomposite showing power density as function of energy density.

The synthesized Pd/NiOPdO nanocomposites were characterized by CV, GCD, and EIS (Fig. 5a–d and 6a–d). The CV and GCD responses of Pd/NiOPdO revealed redox peaks of pseudocapacitor behavior. The presence of redox peaks (0.4 V) at positive current density is the indication of the oxidation process, whereas the cathodic peaks (0.3 V) at the negative current density is the process of reduction. The redox behavior of Pd/NiOPdO was based on the following reaction (M = Ni/Pd).^21,24^Ni^2+^ + 2e^−^ → NiPbO + 2H^+^ + 2e^−^ → Pb^2+^ + H_2_O

However, it was well indicated by XPS that organic functional groups of stabilizing agents (C_8_H_19_N and C_4_H_8_O) were present on the surface of the electrode, which was expected to play their role in the electrochemical reactions of Pd/NiOPdO to enhance the SCs performance. The capacitance of Pd/NiOPdO electrode was calculated at different applied scan rates and shown in Fig. 5c.

A moderate capacitance of 89 F g^−1^ at 2 mV s^−1^ was observed. At a higher scan rate, the specific capacitance value of 50 F g^−1^ was found, and lower capacitance was probably due to higher resistance at higher scan rates which restrict the mobility of H^+^ ion. The electrolyte ions could not reach deep into the active material giving lower capacitance at higher scan rates.^19,21,24^ The specific capacitance (*C*_s_) was also calculated using the GCD method (Fig. 5b–d). The nonlinear response of GCD is well consistent with the pseudocapacitance CV response of Pd/NiOPdO nanocomposite. The highest specific capacitance calculated from the GCD curve was found to be 36 F g^−1^ at 0.5 A g^−1^ (Fig. 5d). The specific capacitance of Pd/NiOPdO nanocomposite at different applied current densities showed 92% capacitance at 1 A g^−1^, 88% retention at 1.5 A g^−1^, and 50% capacitance at 10 A g^−1^ (Fig. 5d). With the increasing scan rate and current density, the specific capacitance was decreased as reported in numerous studies.^19,21,24^ This is because at higher scan rates and current densities electrolyte ions could find enough time to penetrate deep into the active material. Thus, due to the inaccessibility of electrolyte ions to the whole active material the capacitance values decrease within the increasing scan rate and current density. Thus, the CV and GCD response of Pd/NiOPdO nanocomposite electrodes indicate excellent charge storage capacity characteristics of Pd/NiOPdO nanocomposite.

Further, the EIS response of Pd/NiOPdO nanocomposite is measured as shown in Fig. 6a–c. The Nyquist plots showed the semicircle region of charge-transfer-resistance (*R*_ct_) and respective intercept representing internal resistance (*R*_i_) (Fig. 6a and b). In Fig. 6a, the vertical line known as the Warburg element (*Z*_w_) is indicating ideal supercapacitor behaviour.^24^ The magnitude of impedance was decreased as applied AC frequency increased (Fig. 6c) attributed to the conductivity nature of Pd/NiOPdO nanocomposite.

The energy storage capacity of Pd/NiOPdO nanocomposite was evaluated *via* specific energy (W h kg^−1^) and specific power density (W kg^−1^) measurements.^19,21^ The enhanced power density of Pd/NiOPdO nanocomposite was found to be 801.2 W kg^−1^ with a low internal resistance of 0.8 Ω, which was broadly higher than power density of ZnO–activated carbon,^28^ MoO_3_,^25,26^ Co_3_O_4_ nanorods,^27^ CoO/Co_3_O_4_,^29^ and CoO_3_O_4_@carbon^30^ respectively. The Ragone plot of nanocomposite revealed that energy density capacity decreased with the increase of powder density, which is in good agreement with the previous study.^19,21^ The maximum measured power density of Pd/NiOPdO nanocomposite is 802 W kg^−1^ and the maximum calculated energy density is 1.6 W h kg^−1^ at the power density of 144.7 W kg^−1^ as shown in Ragone plot Fig. 6d. The electrochemical energy storage results demonstrated that fabricated Pd/NiOPdO nanocomposite can be a suitable candidate for supercapacitor electrode application.

## 4. Conclusion

In this study, we have successfully synthesized facile Pd/NiOPdO nanocomposites *via* an eco-friendly foliar phyto green method for energy storage supercapacitor application. The synthesized Pd/NiOPdO nanocomposite exhibited nanostructures of the mixed phase of binary metal oxides with Pd. The foliar phyto template-assisted synthesis of Pd/NiOPdO nanocomposite showed that foliar phyto acts as a stabilizing and enhanced electro-active site for the charge storage capacity of the nanocomposite. Although the specific capacitance of Pd/NiOPdO was not significantly higher, however, an increase in power density was observed. The EIS response reveals that fabricated electrode has the potential for supercapacitor energy storage applications.

## Conflicts of interest

The authors declare that they have no known conflict of interest that could have appeared to influence the work reported in this paper.

## Supplementary Material

RA-012-D2RA07292K-s001
