# Supplementary material for: Phyto-synthesized facile Pd/NiOPdO ternary nanocomposite for electrochemical supercapacitor applications
Source: RSC Adv. 2022 Dec 12;12(54):35409–17. doi: 10.1039/d2ra07292k (PMC9742768; doi:10.1039/d2ra07292k)
Supplement: RA-012-D2RA07292K-s001 [file RA-012-D2RA07292K-s001.pdf]

Electronic Supplementary Material (ESI) for RSC Advances.
This journal is © The Royal Society of Chemistry 2022

# Supplementary Information

# Phyto-Synthesized Facile Pd/NiOPdO Ternary Nanocomposite for Electrochemical Supercapacitor Applications

**Irum Shaheen[a,d], Khuram Shahzad Ahmad[a]* Camila Zequine[b], Ram K. Gupta[b], Andrew G. Thomas[c], Anjum Qureshi,[d] Mohammad Azad Malik[c] and Javed H. Niazi[d]**

[a]Department of Environmental Sciences, Fatima Jinnah Women University, Rawalpindi, Pakistan.

[b]Department of Chemistry, Pittsburg State University, 1701 South Broadway Street Pittsburg, KS 66762, USA.

[c]Department of Materials, Photon Science Institute and Sir Henry Royce Institute, Alan Turing Building, University of Manchester, Oxford Road, Manchester M13 9PL, U.K.

[d]Sabanci University, SUNUM Nanotechnology Research and Application Center, Orta Mah. Tuzla 34956 Istanbul, Turkey.

*Email: chemist.phd33@yahoo.com; dr.k.s.ahmad@fjwu.edu.pk.

## Electrochemical Measurement

Electrochemical characterizations of synthesized nanomaterial were carried out using three electrode system VersaSTAT 4-500 electrochemical workstation (Princeton Applied Research, USA).

**Cyclicvoltammetry (CV):** CV was performed by cycling the potential of fabricated electrodes and then measured the corresponding current densities. The purpose of CV was to monitor the redox (oxidation-reduction) behavior of the electrode material within an applied potential range. In the present study, CV analysis of all fabricated electrodes was carried out at scan rate ranged from 2-300 mV/s and at potential window of 0-0.6 V. The current at the prepared electrodes was monitored as a triangular excitation potential was applied . The integrated area of CV curve is significantly important as it reflects the capacitance of the electrode. The

specific capacitance ($C_{sp}$) of the fabricated electrodes was calculated using following relation. [5-6,24]

$$C_{sp} = \frac{\int IdV}{v.\,\Delta V\,.A} \quad ....(1)$$

Where, $\int IdV$ is integral area of CV curve, *v is* scan rate, ΔV is the potential window and A is active area of electrode material on the electrode (or mass of active material on electrode).

**Galvanostatic charge–discharge (GCD):** Galvanostatic charge discharge (GCD) was also used to study the behavior of electrodes at different current densities. GCD was used to calculate the capacitance of the prepared electrodes. [24] The GCD profile for the investigated electrodes was recorded at the applied current density ranged from 0.5-30 A/g. At each current density $C_{sp}$ was calculated using the following equation. [5-6, 19,24]

$$C_{sp} = \frac{I \times \Delta t}{\Delta V \times m} \quad ....2$$

Where, I is the discharge current (A), Δt is the discharge time (s), ΔV is the potential window (V), and m is the mass (g) of the electrodes.

**Energy density (E) and power density (P):** In order to calculate energy density in the three electrode system following equations were used for making an theoretical 2 electrode symmetric configuration. Based on GCD data, energy density (E) and power density (P) of fabricated electrode were estimated using discharge voltage and the discharge capacitance by following equations. [5-6]

$$E = \frac{C\Delta V2}{7.2} \quad ...3$$

$$P = \frac{E(3600)}{t} \quad ...4$$

Where, C (F/g) is capacitance via galvanostatic charge discharge, ΔV (V) is the potential window and t (s) is the discharge time. E and P are expressed as W h/Kg and W/Kg in the present study.
